# Design of a robust and strong-acid MOF platform for selective ammonium recovery and proton conductivity†

**DOI:** 10.1039/d3sc02743k

**Published:** 2023-08-01

**Authors:** Genki Hatakeyama, Hongyao Zhou, Takashi Kikuchi, Masaki Nishio, Kouki Oka, Masaaki Sadakiyo, Yusuke Nishiyama, Teppei Yamada

**Affiliations:** a Division of Chemistry, Graduate School of Science, The University of Tokyo 7-3-1 Hongo Bunkyo-ku Tokyo 113-0033 Japan teppei@chem.s.u-tokyo.ac.jp; b Rigaku Corporation 3-9-12 Matsubaracho Akishima Tokyo 196-8666 Japan; c Department of Applied Chemistry, Faculty of Science Division I, Tokyo University of Science 1-3 Kagurazaka Shinjuku-ku Tokyo 162-8601 Japan; d Nano-Crystallography Unit, RIKEN-JEOL Collaboration Center, RIKEN Yokohama Kanagawa 230-0045 Japan; e JEOL RESONANCE Inc. Akishima Tokyo 196-8558 Japan

## Abstract

Metal–organic frameworks (MOFs) are potential candidates for the platform of the solid acid; however, no MOF has been reported that has both aqueous ammonium stability and a strong acid site. This manuscript reports a highly stable MOF with a cation exchange site synthesized by the reaction between zirconium and mellitic acid under a high concentration of ammonium cations (NH_4_^+^). Single-crystal XRD analysis of the MOF revealed the presence of four free carboxyl groups of the mellitic acid ligand, and the high first association constant (p*K*_a1_) of one of the carboxyl groups acts as a monovalent ion-exchanging site. NH_4_^+^ in the MOF can be reversibly exchanged with proton (H^+^), sodium (Na^+^), and potassium (K^+^) cations in an aqueous solution. Moreover, the uniform nanospace of the MOF provides the acid site for selective NH_4_^+^ recovery from the aqueous mixture of NH_4_^+^ and Na^+^, which could solve the global nitrogen cycle problem. The solid acid nature of the MOF also results in the proton conductivity reaching 1.34 × 10^−3^ S cm^−1^ at 55 °C by ion exchange from NH_4_^+^ to H^+^.

## Abstract 2

A MOF composed of zirconium and melitic acid have acidic sites and exhibit high robustness. It can exchange cation and can selectively capture NH_4_^+^ rather than Na^+^.

## Graphical abstract

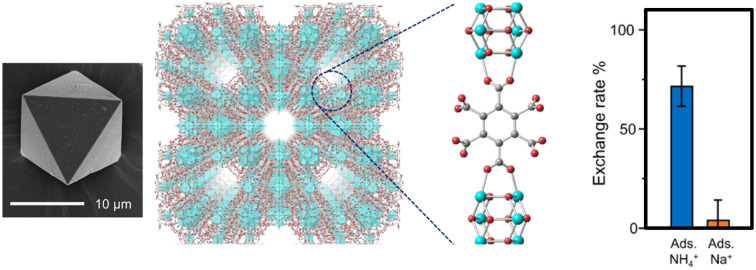

## 1. Introduction

Metal–organic frameworks (MOFs or porous coordination polymers, PCPs) have achieved great progress in the last two decades.^1–8^ Due to the designability and uniform nanopore structure, MOFs show high selectivity for gaseous molecules, which is advantageous for various applications such as gas storage/separation, catalysis, and drug delivery systems.^9–17^ Recently, ion-conductive MOFs have been receiving more attention in accordance with the increasing demand for the development of solid-state electrolytes and energy storage materials.^18–21^ Historically, the acidic points of solid materials have played important roles in ion-conductive materials by providing dissociable ions at the acidic point. These solid acids have been utilized not only as ion conductors but also as ion-exchange materials and solid acid catalysts in industrial processes.^22–25^

Many MOFs are reported to show ionic conductivity, and proton conductive MOFs, in particular, have been intensely studied.^26–28^ However, the number of studies on MOFs as ion-exchange materials is still limited. Some MOFs are reported to adsorb heavy metal ions such as lead, arsenic, selenium, mercury, silver, and palladium,^29–34^ while no MOFs have been reported that can execute reversible ion exchange for small monovalent cations like ammonium (NH_4_^+^), sodium (Na^+^), and potassium (K^+^) in water. The significant barrier for the solid-acid MOF is the poor stability in water.^35^ Early MOFs were very susceptible to decomposition in water or water vapor. Later, many MOFs with improved chemical stability were reported. For example, UiO-66 (ref. ^36^) shows outstanding strength thanks to the strong metal–ligand interaction between the zirconium cation and the carboxy group of terephthalic acid. The combination of the uniform nanopore and ion-exchanging sites of MOFs could expand the scope for the new solid materials endowed with high ionic selectivity and high chemical stability.

Ionic selectivity is vital in the field of radioactive waste treatment or dialysis.^37–40^ In addition, the selective capture of NH_4_^+^ could solve the global nitrogen cycle problem. Today, 1.8 billion tons of ammonia are produced annually by the Haber–Bosch process,^41^ and the accumulation of ammonia in the soil and sea has become a serious issue.^42,43^ The recovery and reuse of NH_4_^+^ in wastewater can reduce the risk of environmental damage. However, the Na^+^ present in wastewater at a high concentration competes with NH_4_^+^ in the ion-exchanging process.^44^ Therefore, the selective recovery of NH_4_^+^ requires an ion-exchange site on the uniform nanoporous frameworks. Highly regulated nanopores of MOFs could be suitable for selective ion capture; however, MOFs with the combination of permanent porosity, high chemical stability and reversible ion-exchanging ability have yet to be achieved.

This paper reports a novel MOF consisting of zirconium and mellitic acid incorporating ammonium cations, hereafter called Zr–mel–NH_4_. This MOF shows high water durability and has a strong-acid point, which can undergo reversible ion exchange with proton (H^+^), NH_4_^+^, Na^+^, and K^+^ cations in an aqueous solution. Furthermore, a high proton conductivity of 1.34 × 10^−3^ S cm^−1^ was achieved by ion-exchanging from NH_4_^+^ to H^+^. Moreover, the uniform pore of the MOF provides selective NH_4_^+^ adsorption from the mixture of NH_4_^+^ and Na^+^.

## 2. Results and discussion

**Fig. 1:**
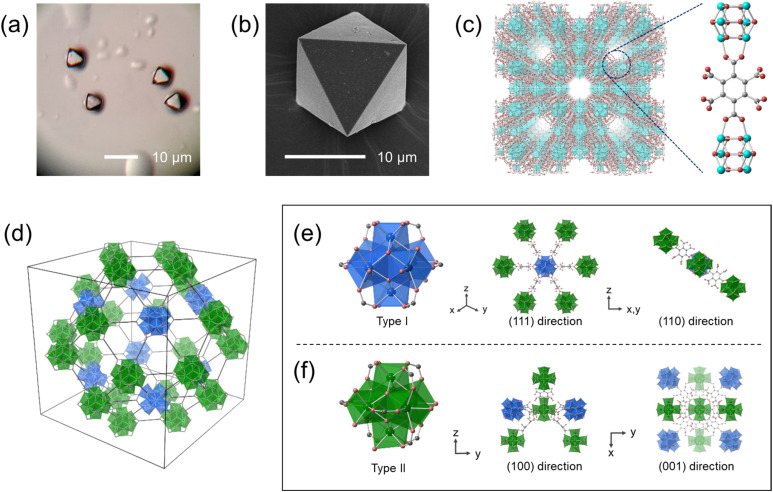
(a) Optical microscope image and (b) SEM image of Zr–mel–NH_4_. (c) Crystal structure of Zr–mel–NH_4_. Blue, red and gray balls show Zr, O and C atoms, respectively. H atoms and water molecules are omitted for clarity. (d) The connection between two types of zirconium clusters within the unit cell. (e) Type I Zr cluster connecting with six type II clusters, viewed from the (111) and (110) directions. (f) Type II Zr cluster connecting with four of both type I and type II clusters, viewed from the (100) and (001) directions.

Hydrothermal synthesis of ZrOCl_2_·8H_2_O (8.3 mM), mellitic acid (0.13 M) with a high concentration of ammonium chloride (2.3 M) and acetic acid (8.7 M) in an aqueous solution produced white crystalline powders. Optical microscope and SEM images show that the crystals have an octahedral shape with a size of 5 to 15 μm (Fig. 1a and b and S1†). The ratio of carbon to nitrogen was found to be 12 : 1 (mol : mol) by elemental analysis (Table S1†), which suggests that Zr–mel–NH_4_ contains one NH_4_^+^ per mellitic acid. The chemical formula was estimated to be Zr_6_O_4_(OH)_4_(L–NH_4_)_3.6_(CH_3_CO_2_)_2.4_·36H_2_O (L = C_12_H_3_O_12_).

We confirmed the structure of Zr–mel–NH_4_ by single-crystal X-ray diffraction analysis (Fig. 1c and Table S2†). The MOF contains Zr_6_O_*x*_(OH)_8−*x*_ clusters bridged by two carboxy groups of the mellitic acid, and the other four carboxy groups remain uncoordinated. The calculated formula agrees with the result of the elemental analysis. Zr–mel–NH_4_ has an *Im*3̄*m* space group with *a* = 41.547(2) Å, which is about twice the cell constant of UiO-66 (*a* = 20.7004(2) Å).^36^ The large cell constant originates from the long-range superlattice structure. The Zr_6_O_4_(OH)_4_ clusters in UiO-66 are connected to the neighboring 12 clusters by the linker. In contrast, Zr–mel–NH_4_ is composed of two types of Zr clusters, type I and type II (Fig. 1d and S2†). The type I cluster connects to six linkers, and the type II cluster is coordinated by eight linkers (Fig. 1e and f). The absence of the linkers caused the periodic lack of Zr clusters and resulted in the superlattice structure.

**Fig. 2:**
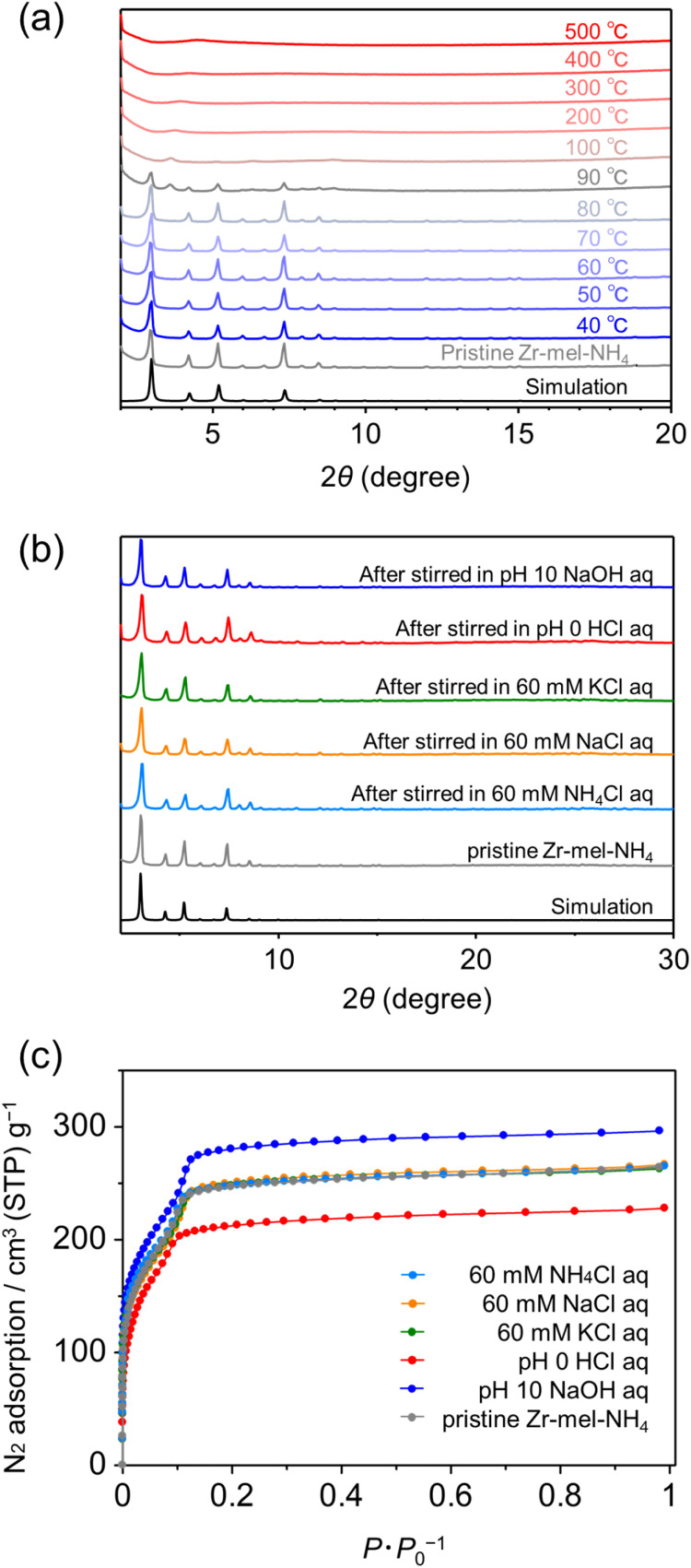
(a) PXRD patterns of Zr–mel–NH_4_ after heating. (b) PXRD patterns and (c) nitrogen gas adsorption isotherms of Zr–mel–NH_4_ after soaking in aqueous 60 mM NH_4_Cl, NaCl, and KCl, pH = 0 HCl, and pH = 10 NaOH solutions.

We examined the thermal stability by thermogravimetric analysis of Zr–mel–NH_4_ and powder XRD patterns of the MOF after heating. The TG curve showed a two-step weight loss at 25 °C and 300 °C corresponding to water elimination and the decomposition of the organic linker in MOFs, respectively (Fig. S3†). PXRD patterns of the Zr–mel–NH_4_ after thermal treatment showed the structural change starting at 90 °C, and the structure became amorphous at 100 °C (Fig. 2a). These results suggest that the structure of Zr–mel–NH_4_ is collapsed by dehydration. The chemical stability of the Zr–mel–NH_4_ was evaluated from the powder XRD patterns after soaking in various aqueous solutions. The PXRD patterns of Zr–mel–NH_4_ after immersion in hydrochloric acid (HCl, pH 0) and sodium hydroxide (NaOH, pH 10) solutions are unchanged from that of the pristine Zr–mel–NH_4_ (Fig. 2b), showing that the structures are highly stable under both acidic and basic conditions. Zr–mel–NH_4_ also has durability with 60 mM NH_4_Cl, NaCl, and KCl aqueous solutions, and the octahedral shape of the MOF crystals was maintained after immersion in these solutions (Fig. S1†). The BET surface area of the Zr–mel–NH_4_ was evaluated by nitrogen (N_2_) gas adsorption analysis (Fig. 2c). Pristine Zr–mel–NH_4_ showed N_2_ uptake up to 0.2*P* × *P*_0_^−1^, and the BET surface area was determined to be 876 m^2^ g^−1^, which is comparable to that of UiO-66 and its derivatives (Table S3†).^45^ Saito–Foley pore size analysis^46,47^ shows that a uniform pore with a cavity size of 7 Å is present in the Zr–mel–NH_4_ (Fig. S4†), which agrees with the cavity size expected from the single-crystal XRD analysis. The MOFs maintained their permanent porosities after soaking in NH_4_Cl, NaCl, and KCl salt solutions (Fig. 2c). It is to be noted that the degradation of porosity is observed for the MOF soaked in HCl, and the capacity was recovered by the additional ion exchange with NH_4_Cl (Fig. S5†). The degradation of the porosity was derived from the collapse of the MOF by the elimination of H_2_O, and the collapse of the MOF was accelerated by the Zr–mel–H. The PXRD patterns are unchanged after the N_2_ gas adsorption experiment (Fig. S6†). As discussed below, the NH_4_ cations in the MOFs soaked in HCl, NaCl, and KCl are thought to be fully replaced by H, Na, and K, respectively.

UiO-66 is widely known for its high stability in water due to the strong coordination bonds with the high-valence zirconium. Therefore, we have attempted to use UiO-66–SO_3_Na, UiO-66–(COOH)_2_, and zirconium-sulfoterephthalate MOF,^48^ and all of them have acid groups capable of trapping NH_4_^+^. However, these MOFs were unstable in an aqueous NH_4_^+^ solution, probably due to the reaction with trace NH_3_ (Fig. S7–S9†). The high stability of Zr–mel–NH_4_ with NH_4_^+^ arises from the synthesis procedure where NH_4_Cl is added to the reaction mixture so that only the MOFs stable with NH_4_^+^ can survive and maintain their framework in the reaction.

**Fig. 3:**
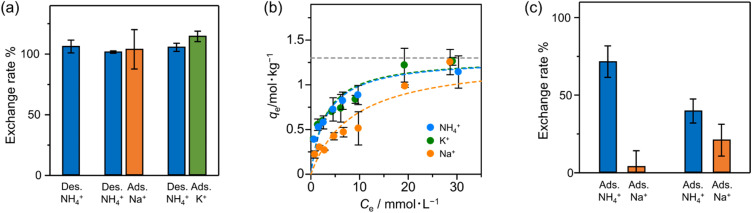
(a) The exchange rate of Zr–mel–NH_4_ to H^+^, Na^+^ and K^+^. (Initial concentration of NaCl and KCl = 30 mM, and pH = 1 hydrochloric acid.) (b) Langmuir adsorption isotherms of Zr–mel–H to NH_4_^+^, Na^+^, and K^+^ (the gray dashed line shows *q*_max_ and is fixed at 1.30 mol kg^−1^). (c) NH_4_^+^/Na^+^ adsorption selectivity of Zr–mel–H.

We investigated the ion-exchanging properties of Zr–mel–NH_4_ with H^+^, Na^+^ and K^+^ (Fig. 3a). The amount of desorbed NH_4_^+^ and the amount of adsorbed exchanging ions were evaluated by ion chromatography, and the result shows that nearly 100% of NH_4_^+^ in Zr–mel–NH_4_ was replaced by H^+^, Na^+^, and K^+^, which is hereafter called Zr–mel–X (X = H, Na, and K) (Table S1†). The existence of NH_4_^+^, Na^+^, and K^+^ in Zr–mel–X was confirmed by SEM-EDX analysis (Fig. S10†). Then, the ion-exchange capability of the Zr–mel–H with NH_4_^+^, Na^+^, and K^+^ was studied. We confirmed that an equimolar amount of the H^+^ on the linker of Zr–mel–H was replaced by NH_4_^+^, Na^+^, and K^+^, respectively (Fig. S11†). The ion exchange between NH_4_^+^ and H^+^ was repeated three times, and the reversibility was confirmed by PXRD patterns and SEM image (Fig. S12–S14†).

To investigate the affinity between Zr–mel–H and each of the cations, the ion-exchange experiments were performed at the initial concentrations of 1, 2, 3, 5, 7, 10, 20, and 30 mmol L^−1^. The Langmuir adsorption isotherms of Zr–mel–H with NH_4_^+^, Na^+^, and K^+^ show greater adsorption amounts with NH_4_^+^ and K^+^ than with Na^+^ below 10 mmol L^−1^ (Fig. 3b). The Langmuir adsorption equation is as follows:1
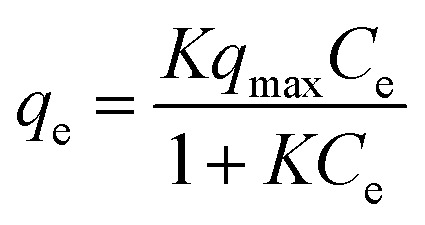
where *q*_e_ is the number of adsorbed cations, *C*_e_ is the adsorbate concentration, respectively. The Langmuir plots show good linearity (Fig. S15†), and the ion-exchange capacity and affinity were evaluated by the fitting of the plots. The maximum capacity (*q*_max_) of Zr–mel–H was fixed at 1.30 mol kg^−1^, which is calculated from the result of elemental analysis. The equilibrium constant (*K*) of Zr–mel–H for NH_4_^+^ and K^+^ is higher than that for Na^+^, showing higher affinity with NH_4_^+^ and K^+^ (Table S4†). Fig. 3c shows the ion-exchanging rate of Zr–mel–H in the mixed solution of NH_4_Cl and NaCl (NH_4_^+^ : Na^+^ = 30 : 30/mM). Notably, 76% of H^+^ on the linkers was exchanged with NH_4_^+^, whereas only 4% of the H^+^ was exchanged with Na^+^. Moreover, the exchanging rate of Zr–mel–H with NH_4_^+^ remains higher than that with Na^+^, even at a higher concentration of Na^+^ than NH_4_^+^ (NH_4_^+^ : Na^+^ = 30 : 120/mM). This high selectivity for NH_4_^+^ is an advantage for the ammonia recovery from wastewater containing a large amount of Na^+^. Note that the concentration of K^+^ in wastewater is generally lower than both NH_4_^+^ and Na^+^, and the little selectivity between NH_4_^+^ and K^+^ should pose less of a problem compared to the competitive adsorption of Na^+^ (Fig. S16†).^49^ The adsorption affinity in water between the guest ions and MOFs is related to the hydration state of the ions. The cations are strongly hydrated in water and become dehydrated when adsorbed by the MOF. The lower dehydration energy required for soft cations like NH_4_^+^ compared to hard cations like Na^+^^50–54^ results in a greater tendency of NH_4_^+^ to be dehydrated and adsorbed inside the MOF. This selectivity is unique to inorganic ion-exchange materials with regulated nanopore structure,^55^ in contrast to the conventional ion-exchanging polymer resins that accommodate the cations under hydrated states.

**Fig. 4:**
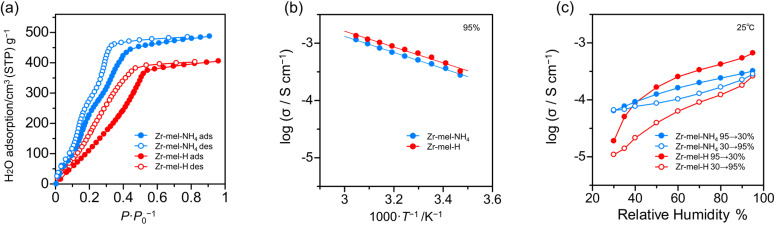
(a) The water vapor adsorption isotherms of Zr–mel–NH_4_ and Zr–mel–H. (b) Temperature dependency and (c) humidity dependency of proton conductivity of Zr–mel–NH_4_ and Zr–mel–H.

The number of acid points, spatially interconnected pores, and ligand defects in solid acids is associated with high proton conductivity.^48,56^ For investigating the relation between proton conductivity and water uptake, the water vapor adsorption analysis of Zr–mel–NH_4_ and Zr–mel–H was executed. From Fig. 4a, Zr–mel–NH_4_ and Zr–mel–H adsorb 487 and 405 cm^3^·(STP) per g at 90% RH, corresponding to 74 and 59 equivalents of water, respectively. Hysteresis between the adsorption and desorption steps was observed in the RH range of 0–50%. Zr–mel–H showed a rise in water adsorption at a higher humidity than that of Zr–mel–NH_4_.

The powders of Zr–mel–NH_4_ and Zr–mel–H were pelletized to perform an AC-impedance analysis (Fig. S17†), and the ionic conductivity (*σ*) was evaluated from the fitting curves of the Nyquist plots (Fig. S18†). The *σ* of Zr–mel–NH_4_ and Zr–mel–H was measured from 15 to 55 °C at 95% RH (Fig. S19†). The *σ* of Zr–mel–NH_4_ and Zr–mel–H were 1.14 × 10^−3^ S cm^−1^ and 1.34 × 10^−3^ S cm^−1^ at 55 °C, respectively (Fig. 4b), which is comparable to the *σ* of a proton conductive MOF with a sulfuric group as the acid point.^57–59^ The activation energy of Zr–mel–NH_4_ and Zr–mel–H shows the same value of 0.12 eV, which indicates that proton transport takes place in a Grotthuss mechanism. The ion exchange from NH_4_^+^ to H^+^ increases the acidity of the acid point and conductivity. Humidity dependency of the resistance was examined by variable humidity impedance measurement from 30 to 95% RH at 25 °C (Fig. S20 and S21†). Two cycles of humidity change were performed to confirm the repeatability. The *σ* of Zr–mel–NH_4_ and Zr–mel–H were 2.83 × 10^−4^ S cm^−1^ and 3.58 × 10^−4^ S cm^−1^ at 95% RH, respectively (Fig. 4c and S22†). The hystereses were observed to be similar to water adsorption isotherms, and their reversibility was also confirmed. Protonated samples have larger hysteresis of conductivity than Zr–mel–NH_4_. This implies that higher water pressure is required to adsorb the water in Zr–mel–H, which is in good agreement with the results of the water vapor adsorption analysis. Zr–mel–NH_4_ and Zr–mel–H maintained their structure after being placed under proton conductivity measurement conditions (Fig. S23 and S24†).

## 3. Conclusions

In summary, we report the first MOF consisting of zirconium and mellitic acid that can reversibly exchange monovalent cations such as H^+^, NH_4_^+^, Na^+^, and K^+^ in water. Single-crystal XRD analysis revealed that Zr–mel–NH_4_ has the structure of UiO-66 with periodic defects and four uncoordinated carboxy groups on the linkers. Elemental analysis indicates that one of them is exchanged with H^+^ and acts as an ion-exchange site. The MOF maintains its structure and permanent porosity stably in acid (pH 0), alkaline (pH 10), and aqueous NH_4_Cl, NaCl, and KCl solutions from PXRD patterns, nitrogen adsorption isotherms, and SEM observations. Zr–mel–NH_4_ exhibits reversible ion-exchange between NH_4_^+^ and H^+^, Na^+^, and K^+^. The ion-exchange site within the uniform nanoporous structure provides selective NH_4_^+^ recovery from a mixture of aqueous NH_4_^+^ and Na^+^ solution, in accordance with the difference in the hydration state of NH_4_^+^ and Na^+^. Furthermore, by ion-exchanging from NH_4_^+^ to H^+^, the proton conductivity reached *ca.* 10^−3^ S cm^−1^ due to the increasing proton donor property. These achievements suggest that Zr–mel–NH_4_ could be applied as a solid acid platform in various applications.

## Author contributions

Teppei Yamada designed the research. Genki Hatakeyama executed all synthesis and measurements and wrote the draft of the manuscript. Takashi Kikuchi executed the SCXRD analysis. Masaaki Sadakiyo conducted the powder XRD measurement and analysis. Yusuke Nishiyama discussed the proton conduction mechanism. Masaki Nishio and Kouki Oka designed the synthetic conditions. Hongyao Zhou and Teppei Yamada revised the manuscript.

## Conflicts of interest

There are no conflicts to declare.

## Supplementary Material

SC-014-D3SC02743K-s001
