# Supplementary material for: Design of a robust and strong-acid MOF platform for selective ammonium recovery and proton conductivity
Source: Chem Sci. 2023 Aug 1;14(34):9068–73. doi: 10.1039/d3sc02743k (PMC10466313; doi:10.1039/d3sc02743k)
Supplement: SC-014-D3SC02743K-s001 [file SC-014-D3SC02743K-s001.pdf]

Electronic Supplementary Material (ESI) for Chemical Science.
This journal is © The Royal Society of Chemistry 2023

Electronic Supplementary Information

Design of a Robust and Strong-Acid MOF Platform for the Selective Ammonium Recovery and Proton Conductivity

Genki Hatakeyama,[a] Hongyao Zhou,[a] Takashi Kikuchi,[b] Masaki Nishio,[a] Kouki Oka,[a] Masaaki Sadakiyo,[c] Yusuke Nishiyama,[d] and Teppei Yamada*[a]

a Division of Chemistry, Graduate School of Science
The University of Tokyo
7-3-1 Hongo, Bunkyo-ku, Tokyo, 113-0033, Japan
E-mail: teppei@chem.s.u-tokyo.ac.jp

b Rigaku Corporation
3-9-12 Matsubaracho, Akishima, Tokyo, 196-8666, Japan

c Department of Applied Chemistry, Faculty of Science Division I
Tokyo University of Science
1-3 Kagurazaka, Shinjuku-ku, Tokyo, 162-8601, Japan

d Nano-Crystallography Unit. RIKEN-JEOL Collaboration Center
RIKEN
Yokohama, Kanagawa, 230-0045, Japan; JEOL RESONANCE Inc., Akishima, Tokyo, 196-8558, Japan

Abstract: Metal–organic frameworks (MOFs) are potential candidates for the platform of the solid acid; however, no MOF has been reported, which has both aqueous ammonium stability and a strong acid site. This manuscript reports a highly stable MOF with a cation exchange site synthesized by the reaction between zirconium and mellitic acid under a high concentration of ammonium cation (NH_4^+). Single-crystal XRD analysis of the MOF revealed the presence of four free carboxyl groups of the mellitic acid ligand, and the high first association constant (pK_{a1}) of one of the carboxyl groups acts as a monovalent ion-exchanging site. NH_4^+ in the MOF can be reversibly exchanged with proton (H^+), sodium (Na^+), and potassium (K^+) cations in an aqueous solution. Moreover, the uniform nanospace of the MOF provides the acid site for a selective NH_4^+ recovery from the aqueous mixture of NH_4^+ and Na^+, which could be beneficial to the global nitrogen cycle problem. The solid acid nature of the MOF also results in the proton conductivity up to 1.34×10^{-3} S·cm^{-1} at 55 °C by its ion exchange from NH_4^+ to H^+.

Table of Contents

1. Synthesis ······ **3**

2. Elemental Analysis ······ **3**

3. SEM images ······ **4**

4. Single-Crystal XRD Analysis ······ **5**

Crystallographic data ······ 5

Comparison of the structure between UiO-66 and Zr-mel-NH_4 ······ 6

5. Thermogravimetric Analysis ······ **7**

6. Nitrogen Gas Adsorption Analysis ······ **8**

Saito–Foley pore size analysis ······ 8

BET surface area ······ 9

PXRD patterns after nitrogen gas adsorption experiment ······ 10

7. Screening of NH_4^+ Stability for Various MOFs ······ **11**

UiO-66-$(COOH)_2$ ······ 11

UiO-66-SO_3Na ······ 12

Zirconium sulfoterephthalate MOF ······ 12

8. SEM-EDX Analysis ······ **13**

9. Ion-Exchange Study ······ **14**

Exchange rate from H^+ to X (X = NH_4^+, Na^+, and K^+) ······ 14

Cycle test between H^+ and NH_4^+ ······ 14

PXRD patterns after cycle test ······ 15

SEM image of the sample after cycle test ······ 15

Equilibrium constant of Zr-mel-H for X (X = NH_4^+, Na^+, and K^+) ······ 16

NH_4^+/K^+ adsorption selectivity ······ 16

10. Proton Conductivity ······ **17**

Measurement set up ······ 17

Equivalent circuit fitting ······ 18

Temperature dependency of Nyquist plot ······ 18

Humidity dependency of Nyquist plot ······ 19

Humidity dependency of proton conductivity ······ 21

PXRD patterns after placed under proton conductivity measurement condition ······ 22

1. Synthesis

$ZrOCl_2·8H_2O$, ammonium chloride, sodium chloride, potassium chloride, and acetic acid and acetic acid were purchased from FUJIFILM Wako Chemicals. Mellitic acid was purchased from Tokyo Chemical Industry. Concentrated hydrochloric acid was purchased from Kanto Chemical Co., Inc.

A solution of $ZrOCl_2·8H_2O$ (129 mg, 0.40 mmol) in 24 mL of acetic acid was added to a solution of mellitic acid (2053 mg, 6.0 mmol) and ammonium chloride (5930 mg, 111 mmol) in 24 mL of water. The mixture was heated at 80 °C for 72 hours by hydrothermal synthesis to obtain a white crystalline powder. The as-synthesized powder was then washed five times with distilled water to remove unreacted reagent, washed with EtOH and ether, and then dried in vacuum at room temperature.
The single crystal was obtained by using similar methods. A solution of $ZrOCl_2·8H_2O$ (32 mg, 0.10 mmol) in 10 mL of acetic acid was added to a suspension of mellitic acid (513 mg, 1.5 mmol) and ammonium chloride (1200 mg, 22 mmol) in 2 mL of water. The mixture was heated at 100 °C for a week by hydrothermal synthesis to obtain a white crystalline powder.

2. Elemental Analysis

Table S1. The result of elemental analysis of Zr-mel-X (X = NH_4, H, Na, K)

	Chemical Formula	C: calc (obs)	H: calc (obs)	N: calc (obs)
Zr-mel-NH_4	$Zr_6O_4(OH)_4$ $(L\text{-}NH_4)_{3.60}$ $(CH_3CO_2)_{2.4}$ $36H_2O$	20.90 (20.75)	3.97 (3.55)	1.83 (1.86)
Zr-mel-H	$Zr_6O_4(OH)_4$ $(L\text{-}H)_{3.55}$ $(L\text{-}NH_4)_{0.05}$ $(CH_3CO_2)_{2.4}$ $24H_2O$	22.07 (21.97)	2.85 (2.87)	0.03 (0.28)
Zr-mel-Na	$Zr_6O_4(OH)_4$ $(L\text{-}Na)_{2.98}$ $(L\text{-}NH_4)_{0.62}$ $(CH_3CO_2)_{2.4}$ $42H_2O$	20.03 (20.03)	3.80 (3.48)	0.30 (0.40)
Zr-mel-K	$Zr_6O_4(OH)_4$ $(L\text{-}K)_{3.43}$ $(L\text{-}NH_4)_{0.17}$ $(CH_3CO_2)_{2.4}$ $42H_2O$	19.64 (19.33)	3.66 (3.15)	0.08 (0.35)

(L-NH_4: $C_{12}H_3O_{12}(NH_4)$, L-H: $C_{12}H_3O_{12}(H)$, L-Na : $C_{12}H_3O_{12}(Na)$, L-K : $C_{12}H_3O_{12}(K)$)

3. SEM Images

SEM observation was performed using a JEOL Ltd. JSM-7500FA. The measurements were carried out at an acceleration voltage of 3 keV and a working distance of 8.0 mm. Samples were placed on carbon tape on an aluminum stage and coated with ~2 nm of osmium.

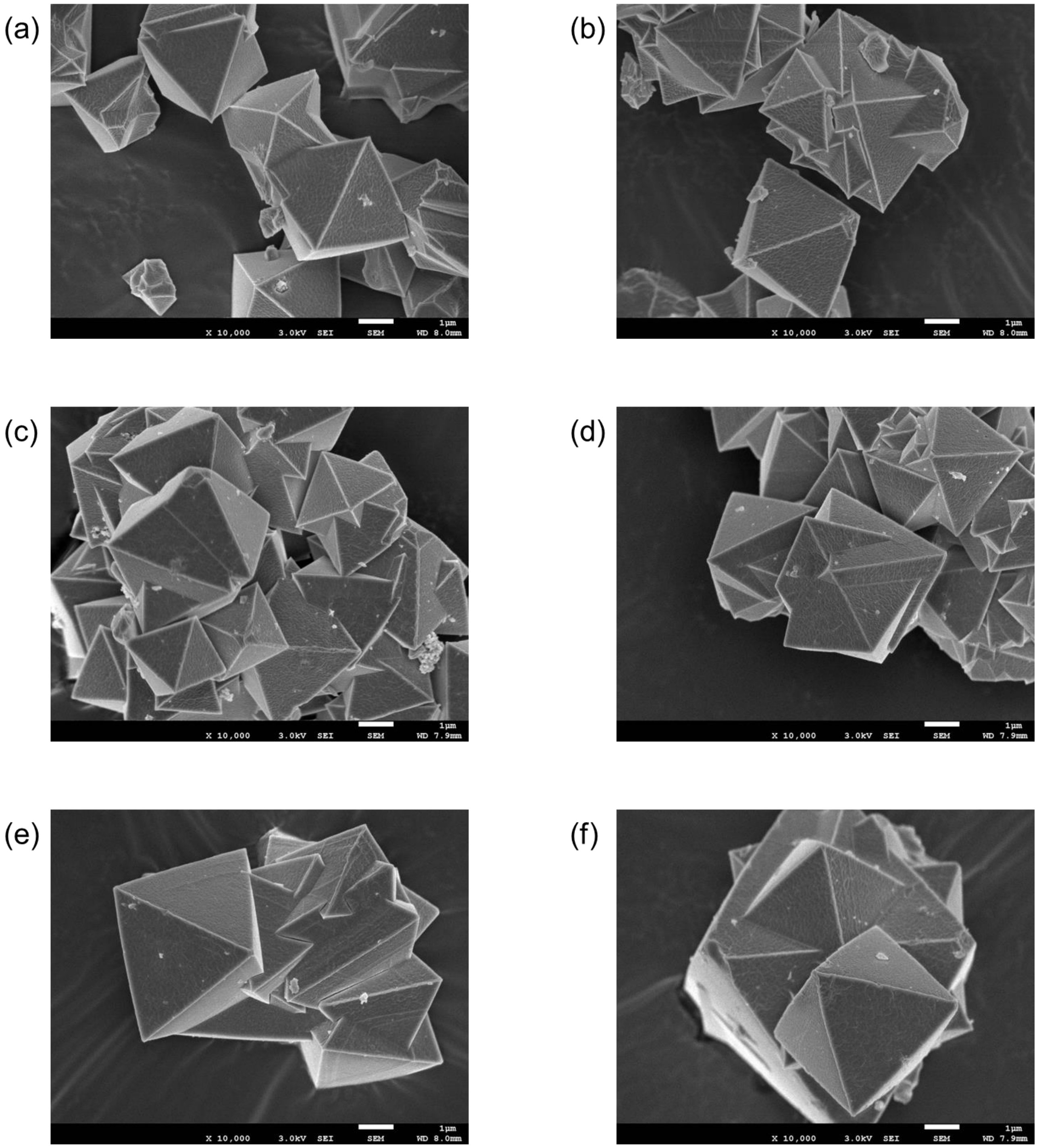

Fig. S1 SEM images of (a) pristine Zr-mel-NH_4 and Zr-mel-NH_4 after soaked in (b) 60 mM NH_4Cl, (c) 60 mM NaCl, (d) 60 mM KCl, (e) pH 0 HCl, and (f) pH 10 NaOH aqueous solutions.

4. Single-Crystal XRD Analysis

Single-crystal XRD data were collected on an XtaLAB PRO P200 (Rigaku Oxford Diffraction) diffractometer equipped with a rotation anode Mo *K*α radiation source (λ = 0.71073 Å), a hybrid pixel array detector (HPAD), and a low-temperature system using cold nitrogen stream. Collected data were integrated, corrected, and scaled by the program CrysAlisPro ver. 1.171.41.119a. Empirical absorption correction was applied in this process. The crystal structure was solved using SHELXT ver. 2018/2 and refined using SHELXL ver. 2018/3 programs.[S1,2]

Table S2. Crystallographic data of Zr-mel-NH_4

Temperature / K	93
Wavelength / Å	0.71069
Formula	$C_{37}O_{57.67}Zr_5$
Formula weight	1823.14
Crystal system	cubic
Space group	*Im*−3*m*
a / Å	41.547(2)
V / Å^3	71714(10)
Z	24
Calculated density / g·cm^{-3}	1.013
θ range	1.834° ~ 29.302°
Crystal form	Octahedral
Crystal color	colorless
Crystal size	0.01×0.01×0.01
h range	−51 ~ 56
k range	−56 ~ 55
l range	−56 ~ 55
Weighting scheme	$w = [\sigma^2(F_o^2)+(0.1485P)^2]$, $P = (F_o^2+2F_c^2)/3$
$F_{(000)}$*	21200
R_1**	8.26%
wR_2***	28.24%
R_{int}	26.47%
GOF****	1.047

*$F_{(000)} = (f_r + f_i)^{1/2}$ where f_r and f_i are the real part and imaginary part of the scattering factors at theta = 0 degrees, respectively.

**$R_1 = (\Sigma||F_o| - |F_c||)/\Sigma|F_o|$ for observed reflections, where F_o and F_c are the observed and calculated normalized factor, respectively.

***$wR_2 = [\{\Sigma(w(F_o^2 - F_c^2)^2)\}/ \Sigma(w(F_o^2)^2)]^{1/2}$ for independent reflections.

****GOF = $[\{\Sigma(w(F_o^2 - F_c^2)^2)\}/(n\text{-}p)]^{1/2}$ where *n* and *p* are the number of the reflection and the parameters in the refinement, respectively.

4. Single-Crystal XRD Analysis (continued)

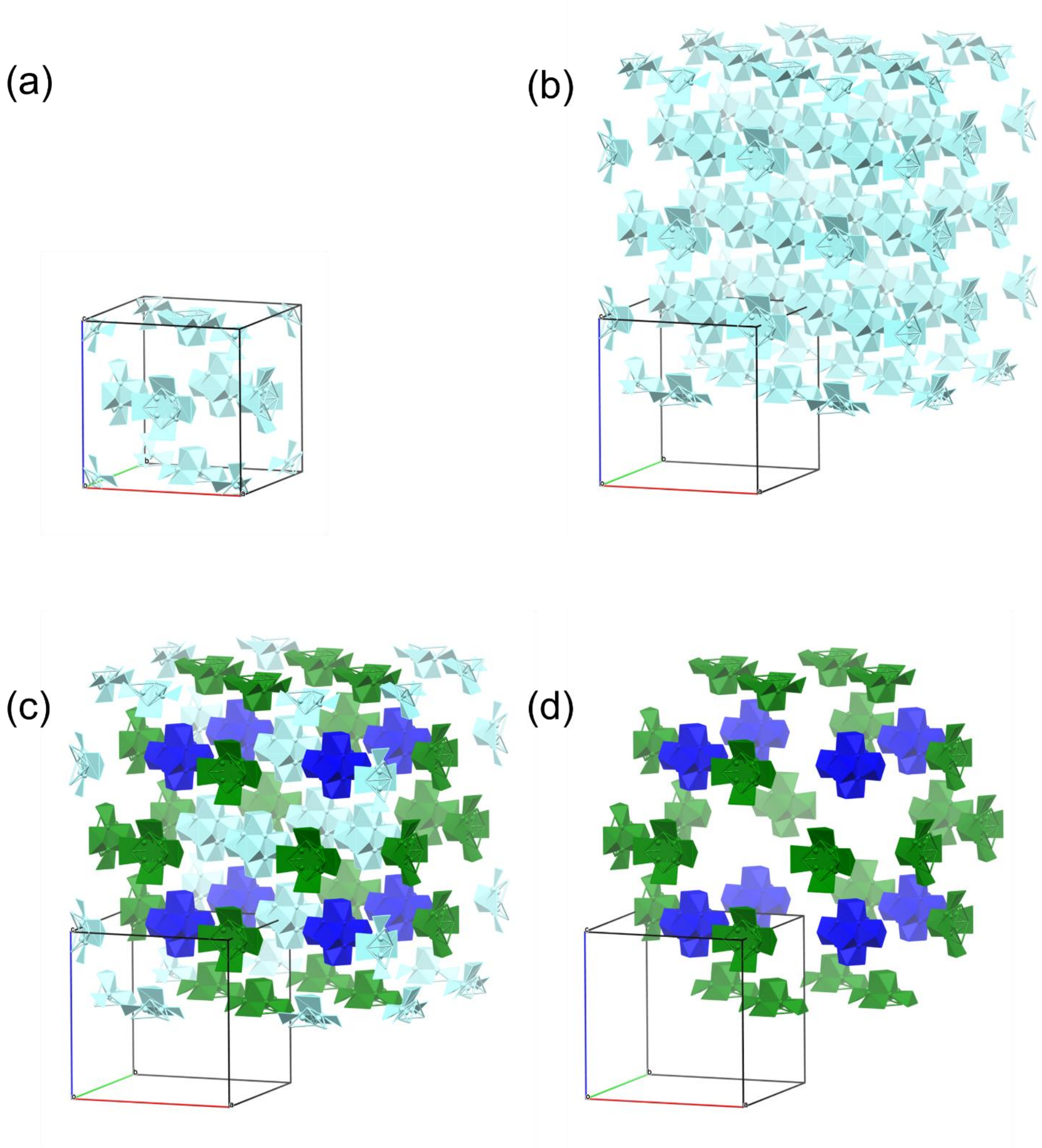

Fig. S2 The crystal structures of UiO-66 and Zr-mel-NH_4. The arrangement of Zr_6O_8 clusters (pale blue) (a) in the unit cell of UiO-66, (b) in the extended lattice of UiO-66 in the range from (0, 0, 0.5) to (2, 2, 2.5). (c) The clusters are classified into Type I (blue), Type II (green) and defects (pale blue) in the Zr-mel-NH_4. The ratio of Type I:Type II:defect is 8:8:12. (d) The arrangement of Zr_6O_8 clusters of Zr-mel-NH_4.

5. Thermogravimetric Analysis

Thermogravimetric analysis was conducted using a DTG-60 (SHIMADZU). The samples in aluminum pans were heated from RT to 500 °C at 5 °C·min^{-1} under nitrogen flow.

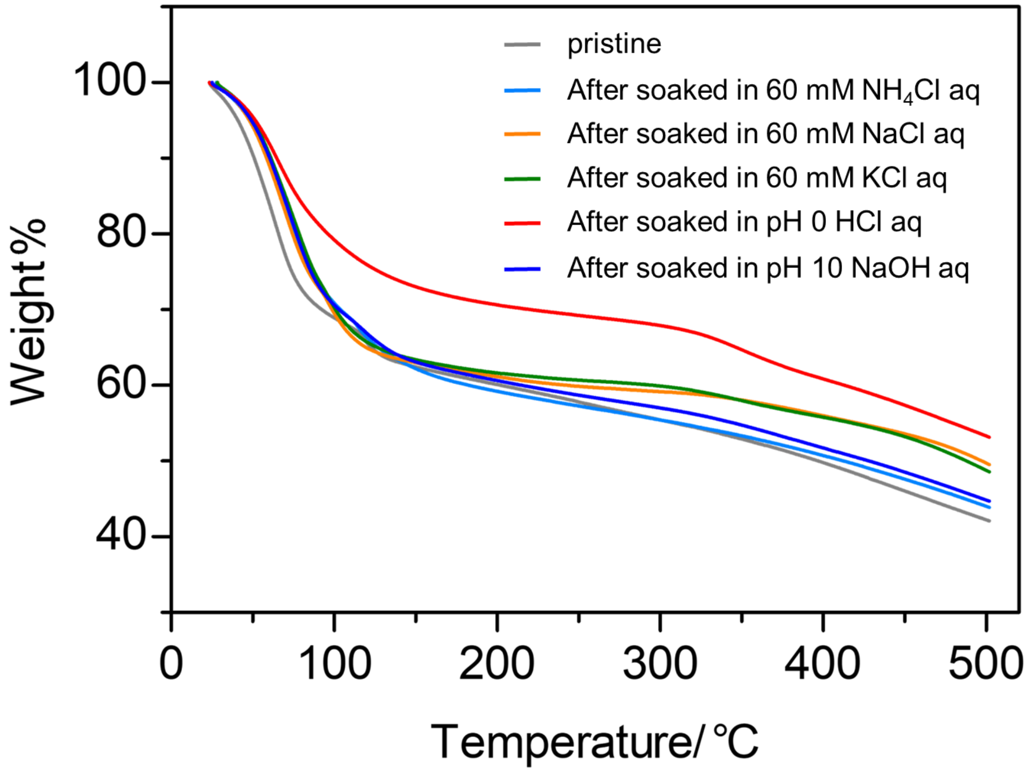

Fig. S3 TG curves of pristine Zr-mel-NH_4 and Zr-mel-NH_4 after soaked in various aqueous solutions.

6. Nitrogen Gas Adsorption Study

Nitrogen gas adsorption analysis was performed by a BELSORP-Max (BEL Japan) using nitrogen gas (≥99.9995%) at 77 K with a liquid nitrogen bath. The samples were activated by reducing pressure at room temperature and purging with nitrogen gas. This process was repeated three times.

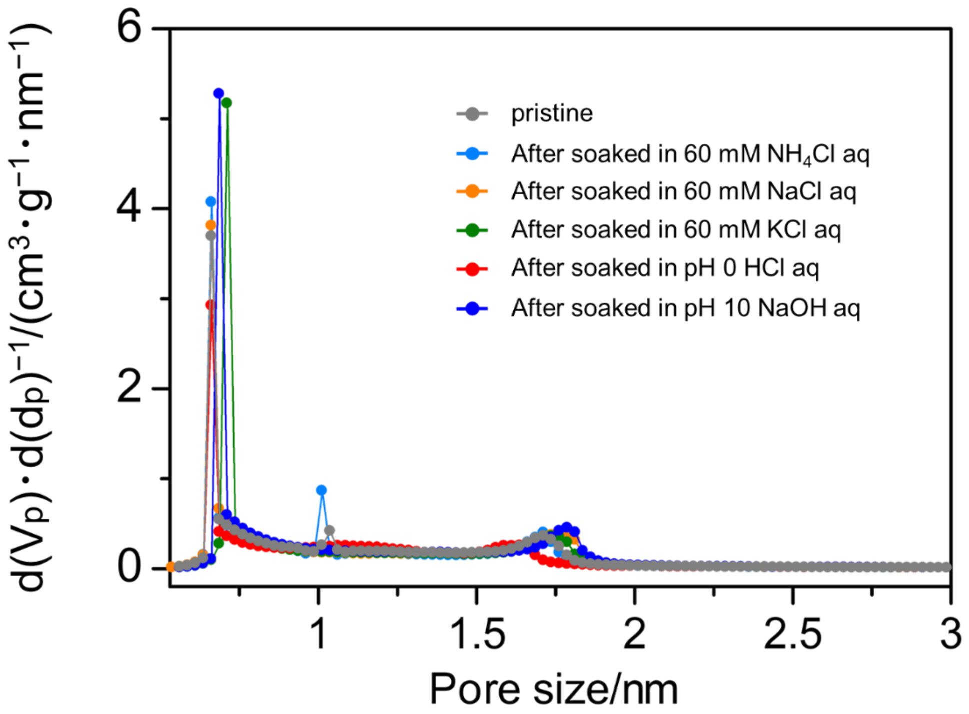

Fig. S4 Saito−Foley pore size analysis of pristine Zr-mel-NH_4 and Zr-mel-NH_4 after soaked in various aqueous solutions.

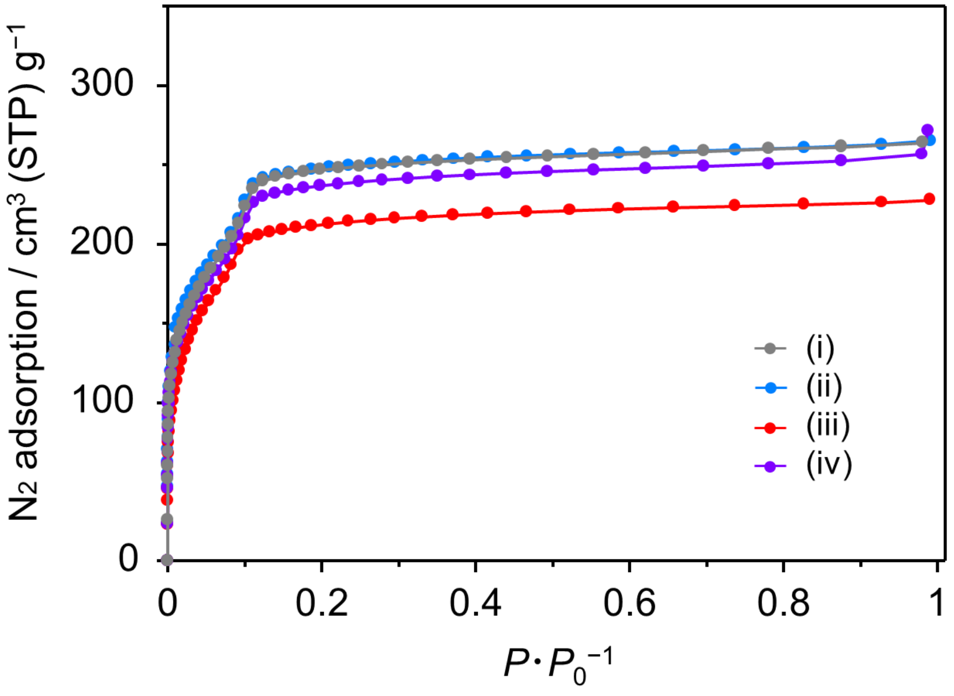

Fig. S5 Nitrogen gas adsorption isotherms of Zr-mel MOFs. (i) pristine Zr-mel-NH4 (grey). (ii) after soaking (i) in 60 mM NH_4Cl (light blue). (iii) (ii) soaked in HCl (pH0) (red). (iv) (iii) soaked in 60 mM NH_4Cl (purple).

Table S3. BET surface area of pristine Zr-mel-NH_4 and Zr-mel-NH_4 after soaked in various aqueous solutions.

Sample	BET surface area / $m^2 \cdot g^{-1}$	Reference
Pristine Zr-mel-NH_4	876	This work
Zr-mel-NH_4 after soaked in 60 mM NH_4Claq	892	
Zr-mel-NH_4 after soaked in 60 mM NaClaq	897	
Zr-mel-NH_4 after soaked in 60 mM KClaq	887	
Zr-mel-NH_4 after soaked in pH 0 HClaq	845	
Zr-mel-NH_4 after soaked in pH 10 NaOHaq	978	
UiO-66	769	Ref[S3]
UiO-66 (solvothermal)	1525	
UiO-66-$(COOH)_2$	491	
UiO-66-$(COOH)_4$	212	

6. PXRD patterns After Nitrogen Gas Adsorption Experiment

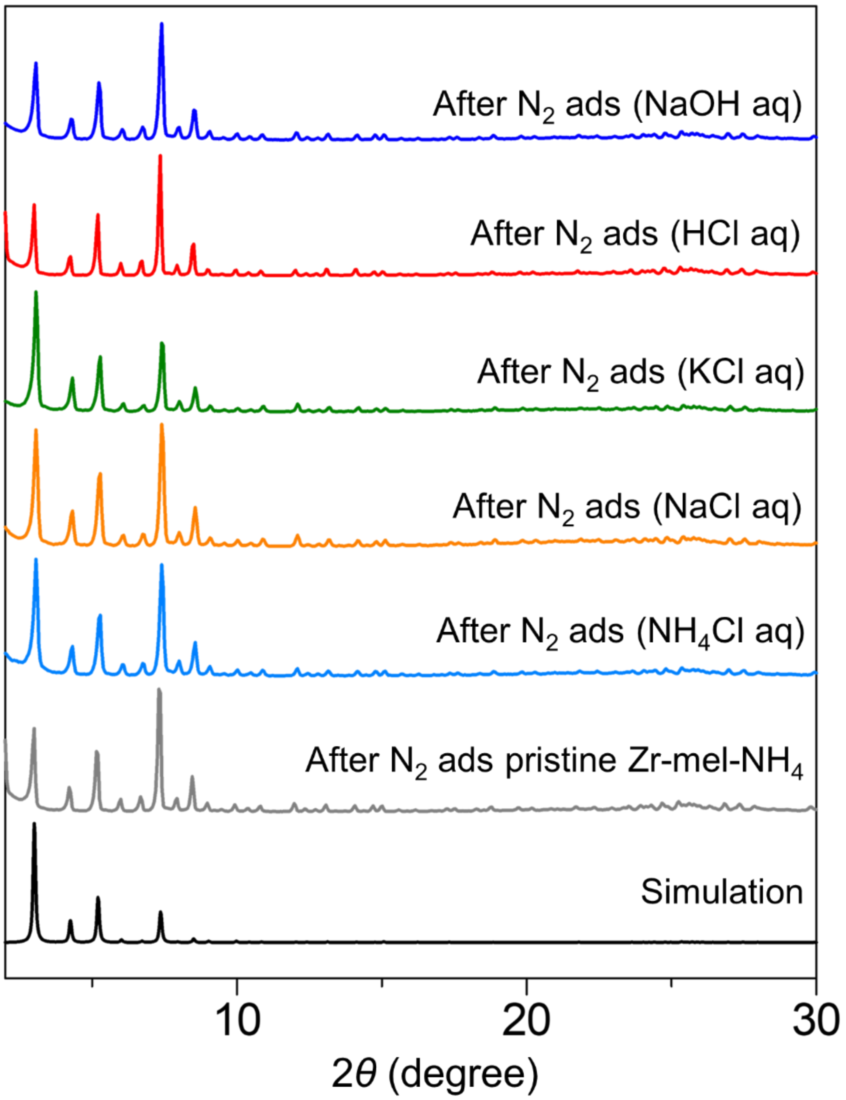

Fig. S6 PXRD patterns of Zr-mel-NH_4 after nitrogen gas adsorption experiment soaked in each aqueous solution.

7. Screening of NH_4^+ Stability for Various MOFs

UiO-66-$(COOH)_2$, UiO-66-SO_3Na, and zirconium sulfoterephthalate MOF were synthesized according to the previous reports[S4–6] and were soaked in an aqueous NH_4Cl solution to evaluate the NH_4^+ stability.

20 mg of UiO-66-$(COOH)_2$ andUiO-66-SO_3Na were soaked in 20 mL of aqueous NH_4Cl solution (30 mM) at 30 °C for 3 h. Zirconium sulfoterephthalate MOF was also immersed in NH_4Cl solution for 1 h under same conditions. Each sample was collected by centrifugation and washed with water to measure the PXRD patterns.

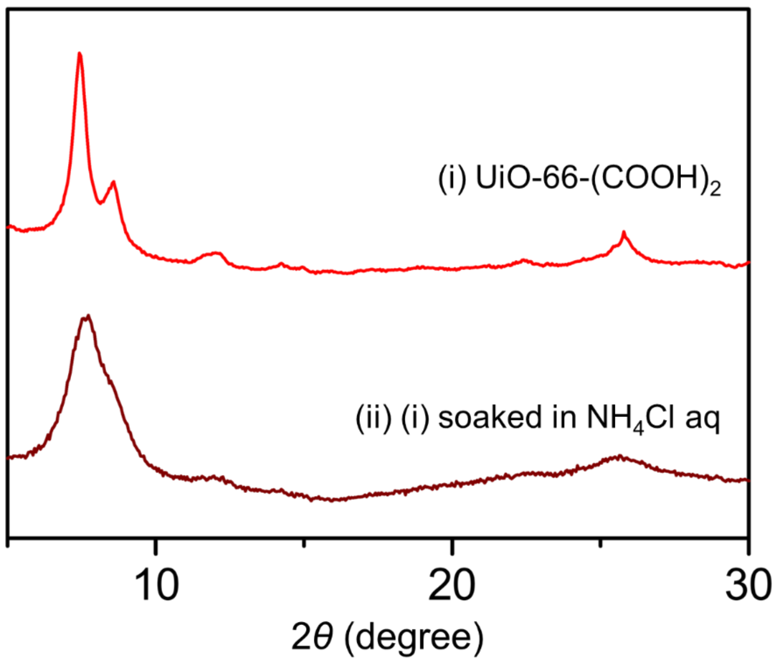

Fig. S7 PXRD patterns of UiO-66-$(COOH)_2$ (i) before and (ii) after soaked in NH_4Cl aqueous solutions.

7. Screening of NH_4^+ Stability for Various MOFs (continued)

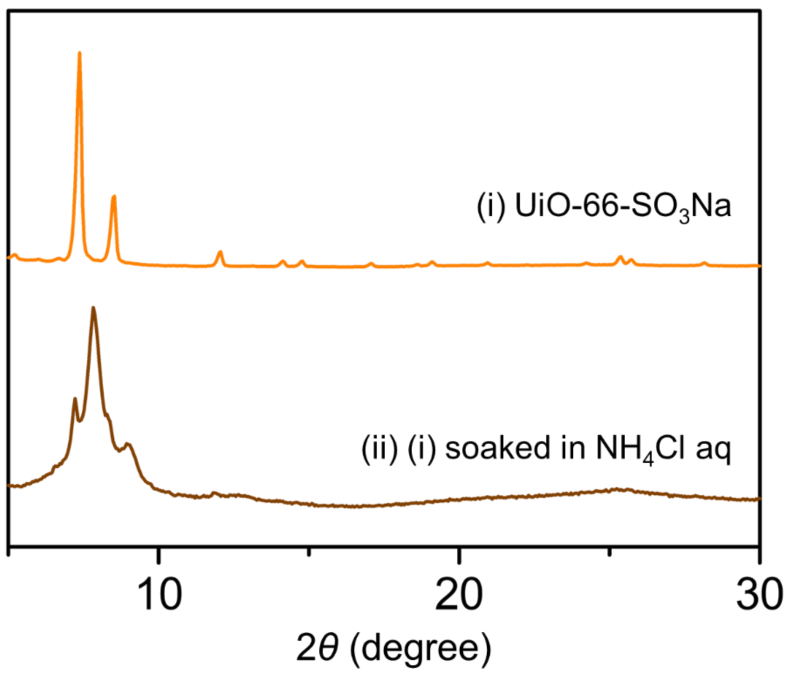

Fig. S8 PXRD patterns of UiO-66-SO_3Na (i) before and (ii) after soaked in NH_4Cl aqueous solutions.

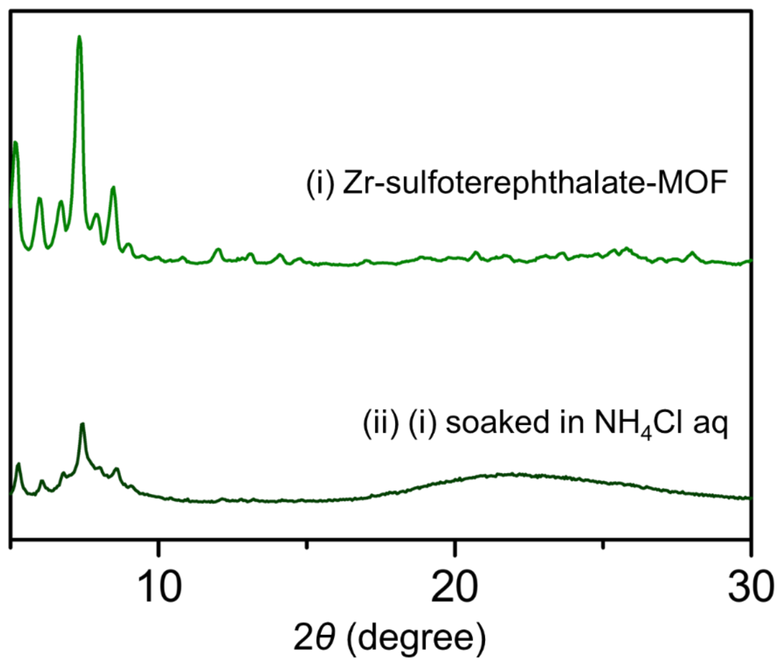

Fig. S9 PXRD patterns of zirconium sulfoterephthalate MOF (i) before and (ii) after soaked in NH_4Cl aqueous solutions.

8. SEM-EDX Analysis

SEM-EDX analysis was performed using a JEOL Ltd. JSM-7800F. The measurements were carried out at an acceleration voltage of 10 keV and a working distance of 10 mm. Samples were placed on carbon tape on an aluminum stage and coated with ~2 nm of osmium.

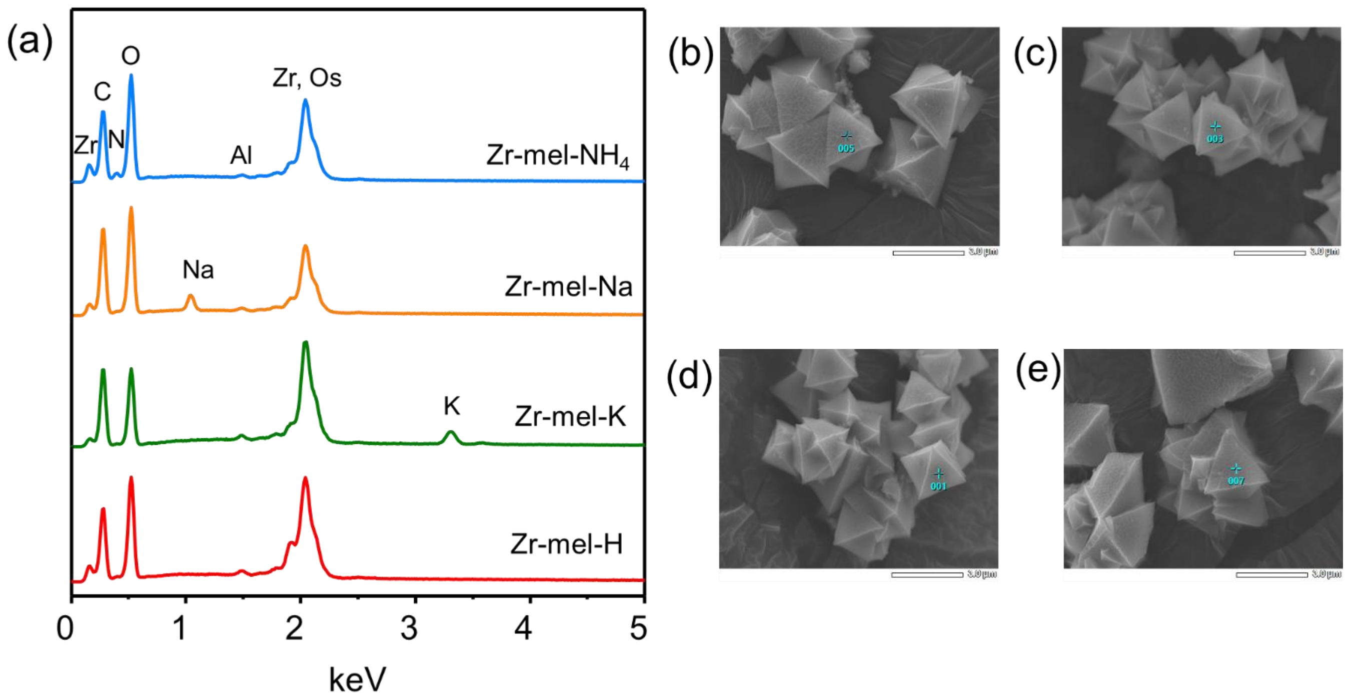

Fig. S10 (a) EDX spectra of Zr-mel-X (X = NH_4, Na, K, and H). The spectra were obtained at the spotted position of (b) Zr-mel-NH_4, (c) Zr-mel-Na, (d) Zr-mel-K, and (e) Zr-mel-H.

9. Ion-Exchange Study

ca. 100 mg of pristine Zr-mel-NH_4 was added to 40 mL (pH 1) of hydrochloric acid. The suspension was stirred at 30 °C for 60 min. After stirring, the supernatant and the powder were separated by centrifugation at 4000 rpm for 10 min before filtration with a 45 µm membrane filter. The amount of released ammonium ion into the solution was evaluated using ion chromatography (IC, 883 Basic IC plus; Metrohm). 20 mg of Zr-mel-H or pristine Zr-mel-NH_4 were added to a 20 mL (30 mM) aqueous solution of ammonium chloride, sodium chloride, potassium chloride, and the mixture of these salt. These suspensions were proceeded with a similar method as above and evaluated for each cation concentration. The reversibility of ion exchange between proton and ammonium cations was investigated by repeating the ion exchange test.

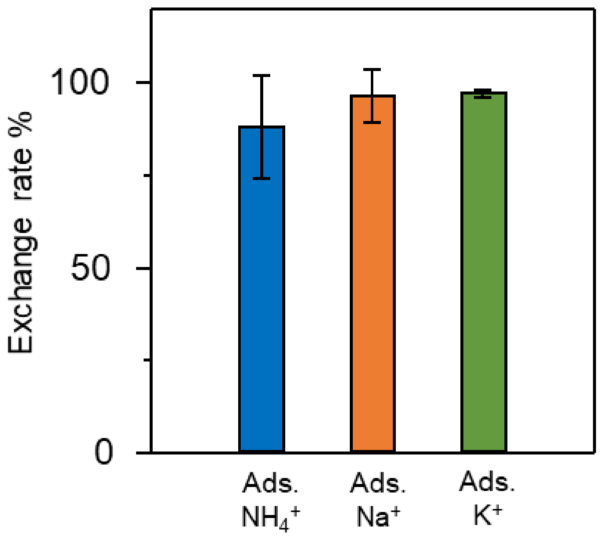

Fig. S11 The exchange rate from H^+ to NH_4^+, Na^+, and K^+. (Initial concentration of NH_4Cl, NaCl, and KCl = 30 mM)

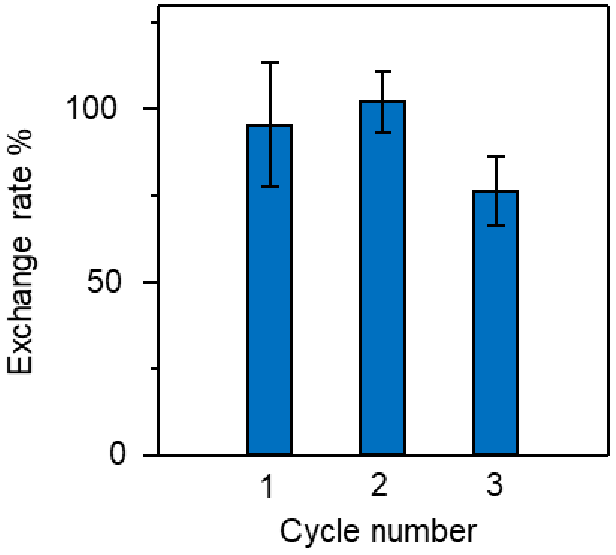

Fig. S12. The repeatability of adsorbing NH_4^+ by Zr-mel-H. (Initial concentration of NH_4Cl = 30 mM, pH 1 HCl)

9. PXRD Patterns After Ion-Exchange Experiment

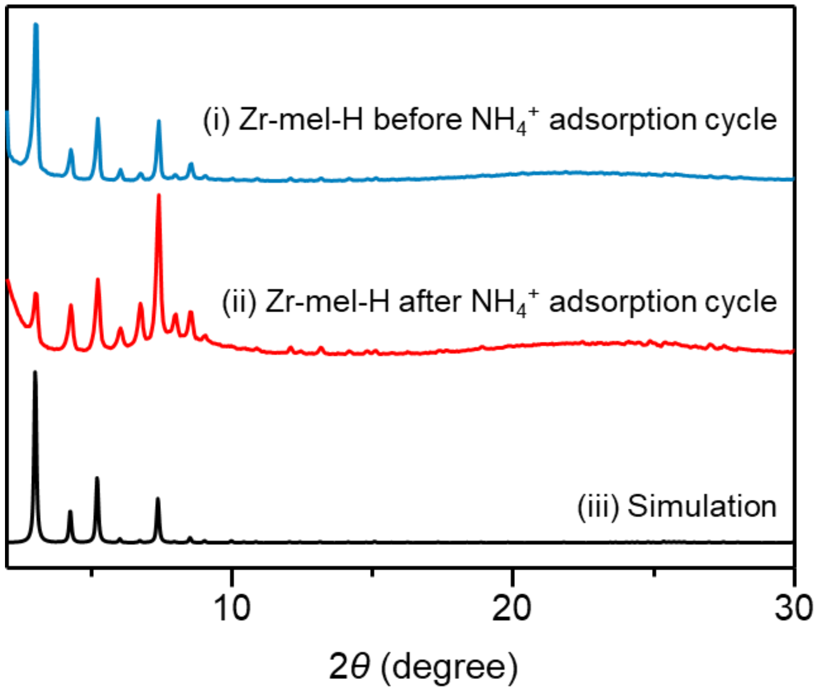

Fig. S13 PXRD patterns of Zr-mel-H after three cycles of NH_4^+ adsorption and desorption. After NH_4 adsorption, the intensity of the first peak in (ii) decreases due to the incorporation of guests, i.e. water, and Y axis of it was magnified. The additional peaks observed in (ii) locates the diffraction angles where the original crystal could have the intensity, indicating change of intensity by the guest introduction.

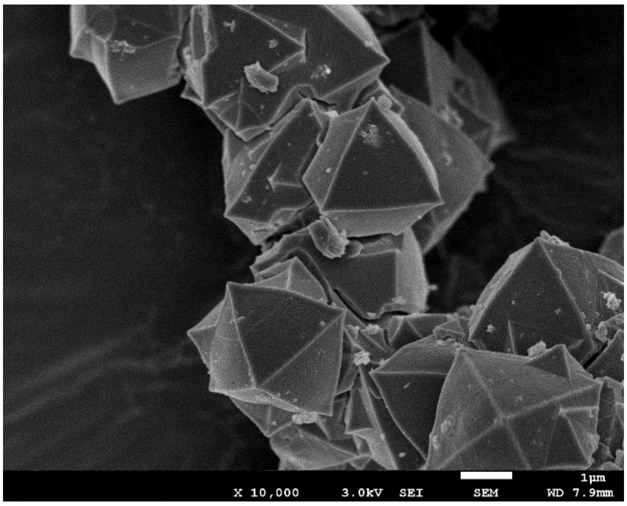

Fig. S14 SEM images of Zr-mel-NH_4 after soaked three times each in pH 1 HCl and 30 mM aqueous NH_4Cl solution.

9. Ion-Exchange Study

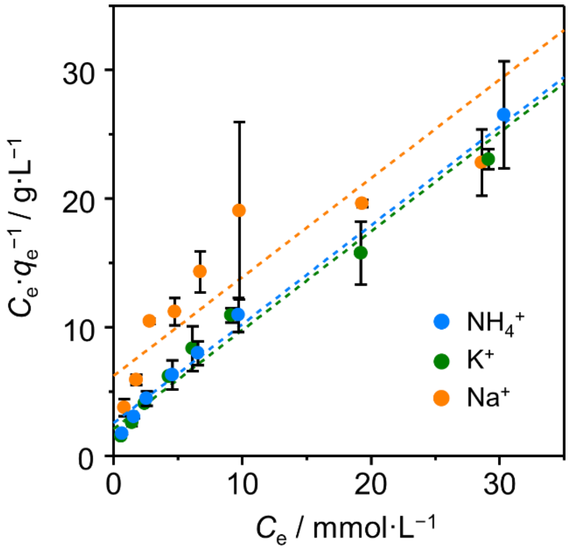

Fig. S15 Langmuir plot for Zr-mel-H to NH_4^+, K^+, and Na^+ cations.

Table S4. The equilibrium constant *K* and coefficient of determination R^2 for Zr-mel-H to various cations in aqueous solutions.

	K/L·mol^{-1}	R^2
NH_4^+	0.32	0.990
Na^+	0.12	0.821
K^+	0.34	0.974

To evaluate the selectivity for NH_4^+ and K^+, Zr-mel-H was soaked in the mixed solution of NH_4Cl and KCl (NH_4^+:Na^+ = 30: 30/mM).

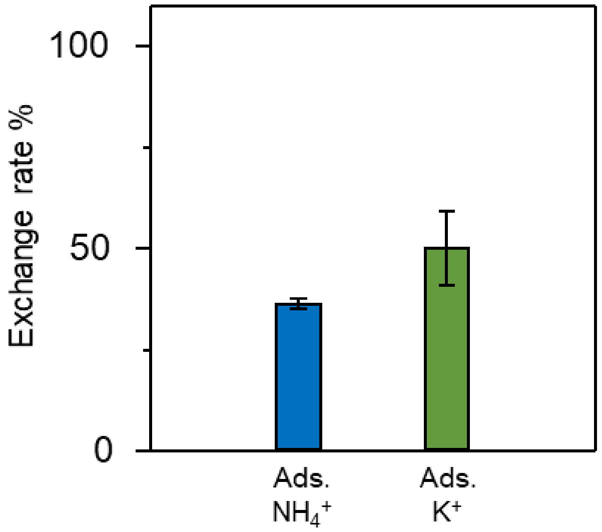

Fig. S16 The exchange rate of ammonium and potassium cations.

10. Proton Conductivity

The powder was pelletized by pressing in a cylindrical die (1 cm diameter, about 1 mm thickness) at ca. 0.3 GPa for 60 seconds. The resistance was estimated from the equivalent circuit fitting of the Nyquist plots. Proton conductivity was calculated with $\sigma = (1/R)\ (t/a)$, where σ is conductivity, R is the resistance inside the MOF, and t and a are the thickness and area of the pellet, respectively. AC impedance measurement was performed using a 1260 Impedance/Gain-Phase Analyzer (Solartron) with a temperature and humidity chamber IW222 (Yamato) from 10 MHz–10 Hz. Humidity dependency from 30 to 95%RH was investigated at 25 °C. Temperature dependency from 15 to 55 °C was investigated at 95%RH. AC impedance was measured after waiting at least 90 min after reaching the target humidity or temperature. Water vapor adsorption analysis was performed at 298 K by a similar setup using ultra-pure water-treated freeze-pump-thaw cycling.

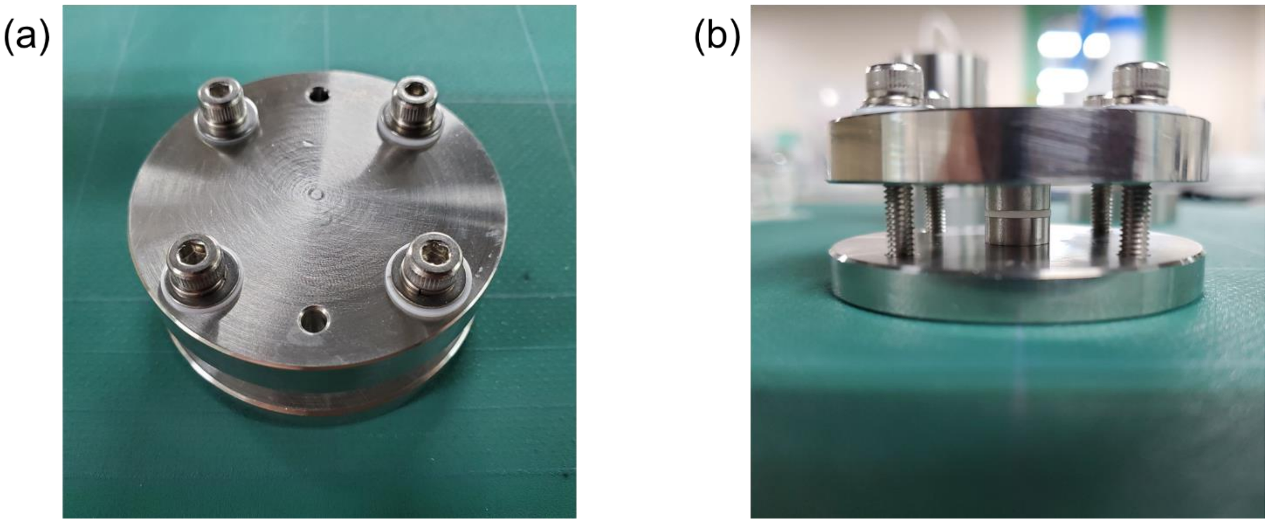

Fig. S17 The measurement cell for the proton conductivity. (a) front view and (b) side view.

10. Proton Conductivity (continued)

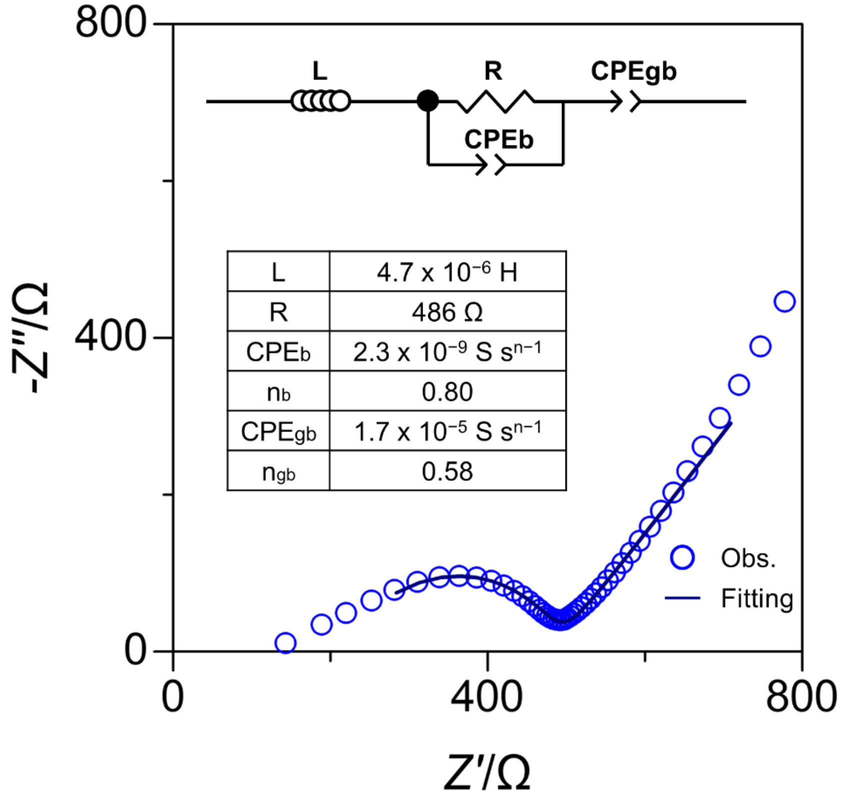

Fig. S18 An example of the equivalent circuit fitting of a Nyquist plot. The data was obtained by AC impedance measurement of Zr-mel-NH_4 at 15 °C and 95%RH.

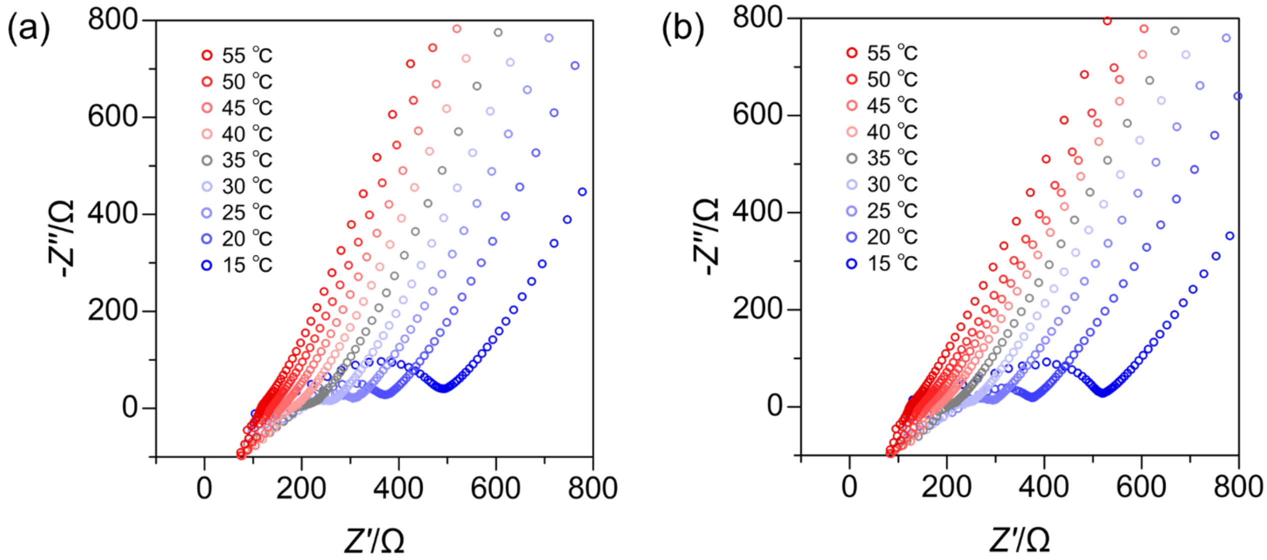

Fig. S19 Nyquist plots of (a) Zr-mel-NH_4 and (b) Zr-mel-H under 95%RH at variable temperature.

10. Proton Conductivity (continued)

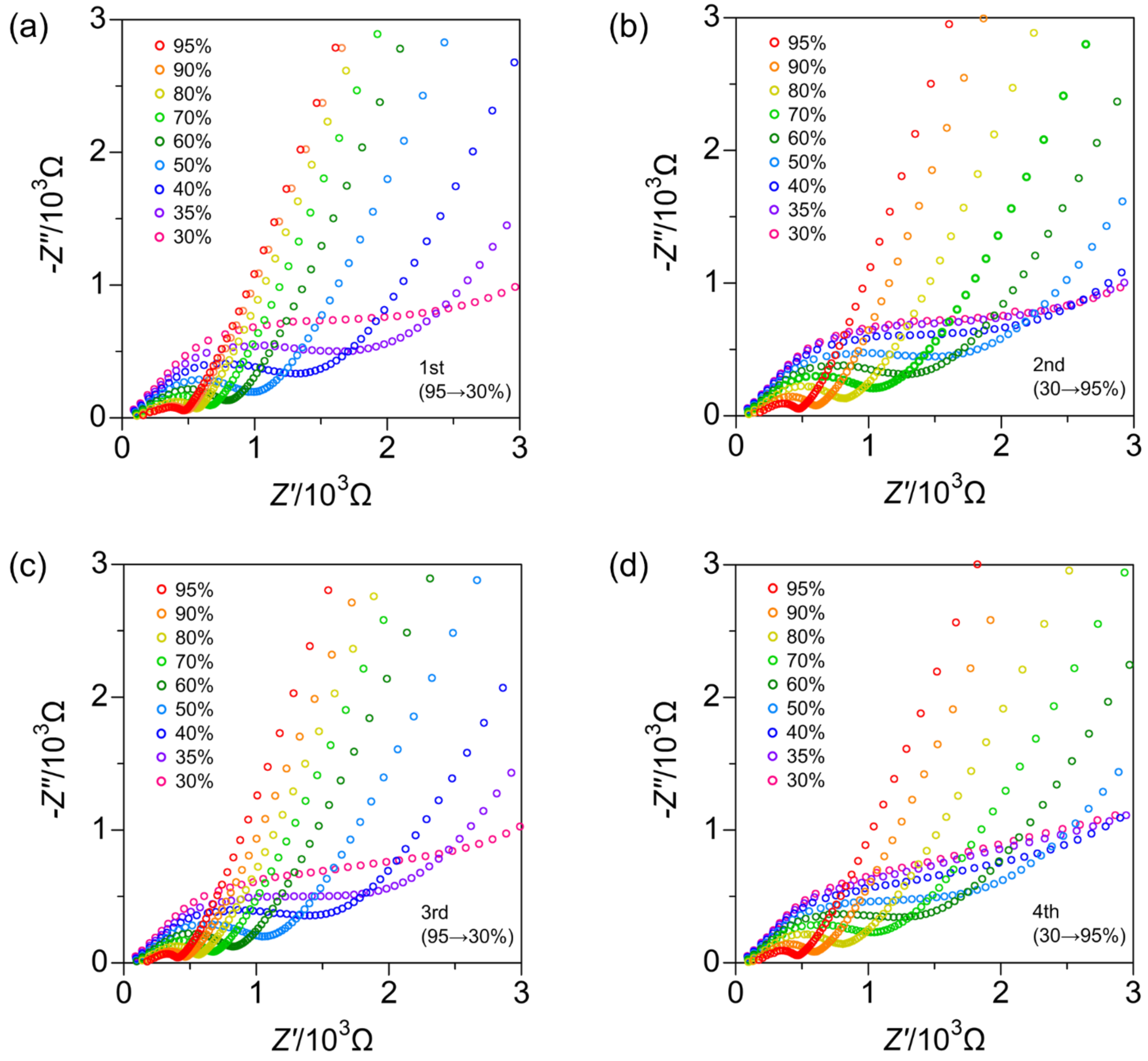

Fig. S20 Nyquist plots of Zr-mel-NH_4 at 25 °C under variable humidity. (a) 1st cycle (from 95 to 30%), (b) 2nd cycle (from 30 to 95%), (c) 3rd cycle (from 95 to 30%), (d) 4th cycle (from 30 to 95%).

10. Proton Conductivity (continued)

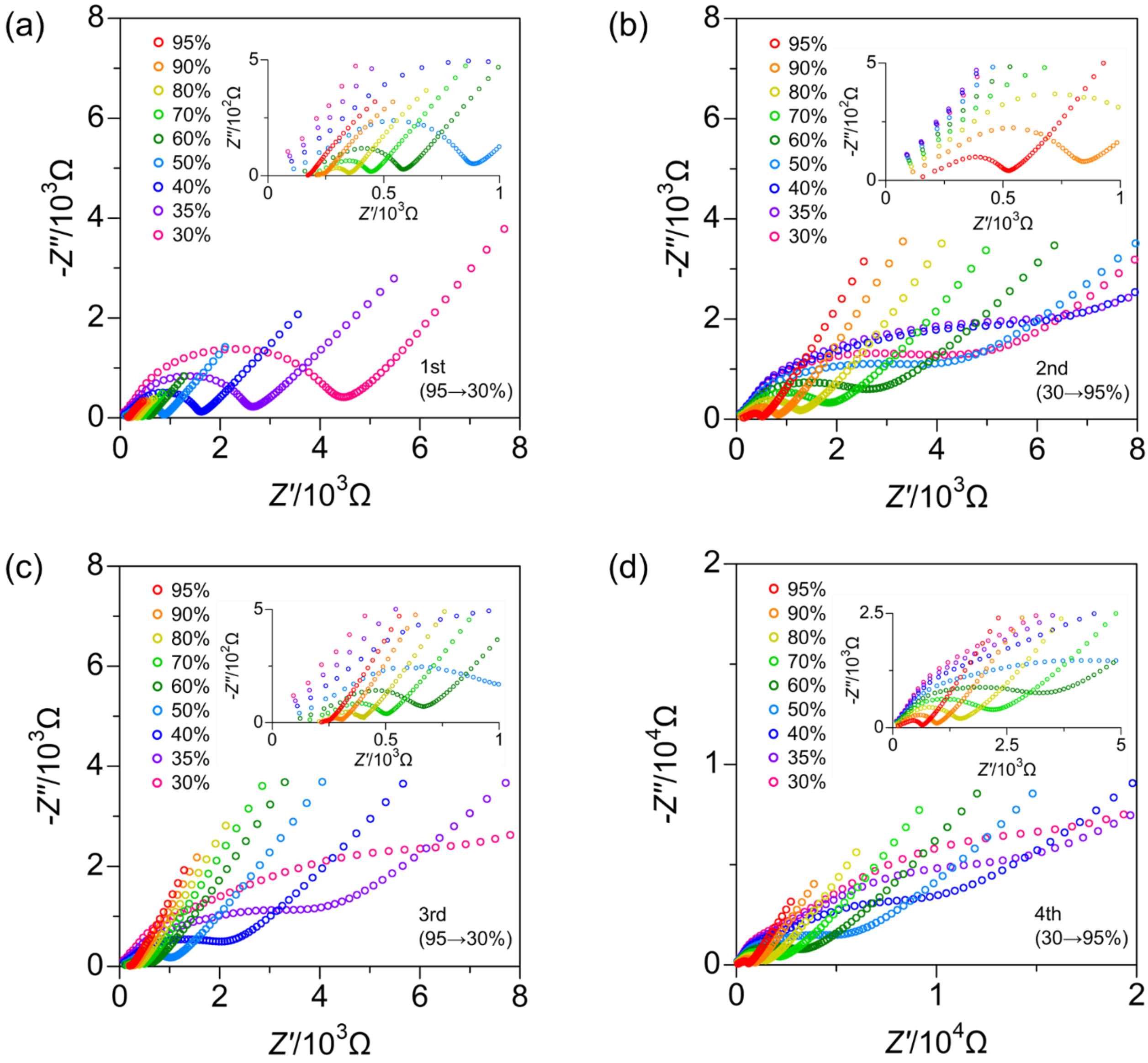

Fig. S21 Nyquist plots of Zr-mel-H at 25 °C under variable humidity. (a) 1st cycle (from 95 to 30%), (b) 2nd cycle (from 30 to 95%), (c) 3rd cycle (from 95 to 30%), (d) 4th cycle (from 30 to 95%).

10. Proton Conductivity (continued)

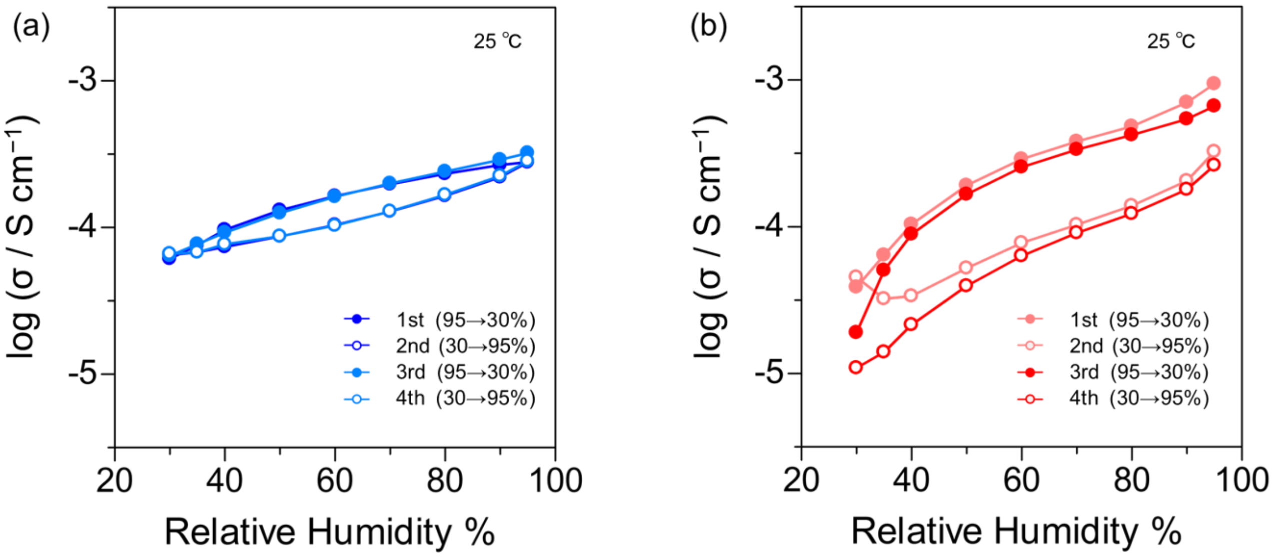

Fig. S22 Humidity dependency of proton conductivity of (a) Zr-mel-NH_4 and (b) Zr-mel-H.

10. PXRD Patterns After Proton Conductivity Measurement Condition

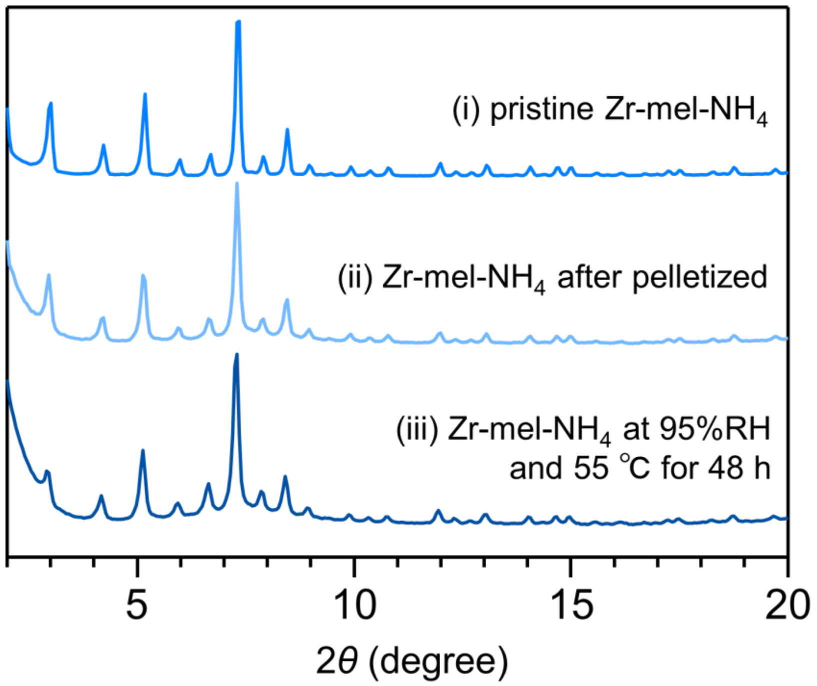

Fig. S23 PXRD patterns of (i) pristine Zr-mel-NH_4 , (ii) Zr-mel-NH_4 after pelletized and (iii) that being placed at proton conductivity measurement condition.

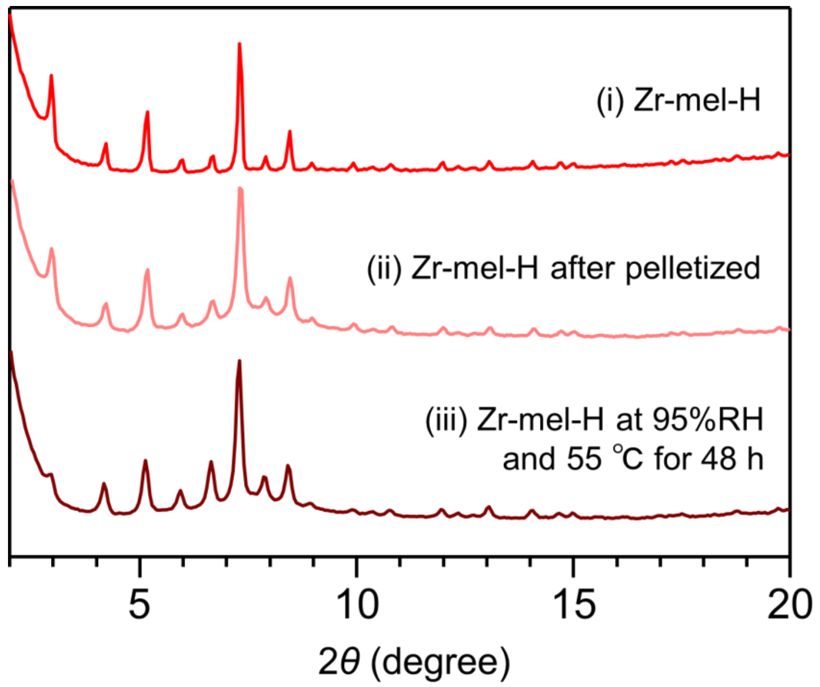

Fig. S24 PXRD patterns of (i) pristine Zr-mel-H , (ii) Zr-mel-H after pelletized and (iii) that being placed at proton conductivity measurement condition

References

S1 G. M. Sheldrick, *Acta Crystallogr. Sect. A Found. Crystallogr.*, 2015, **71**, 3–8.

S2 G. M. Sheldrick, *Acta Crystallogr. Sect. C Struct. Chem.*, 2015, **71**, 3–8.

S3 Z. Hu, Y. Peng, Z. Kang, Y. Qian and D. Zhao, *Inorg. Chem.*, 2015, **54**, 4862–4868.

S4 Q. Yang, S. Vaesen, F. Ragon, A. D. Wiersum, D. Wu, A. Lago, T. Devic, C. Martineau, F. Taulelle, P. L. Llewellyn, H. Jobic, C. Zhong, C. Serre, G. De Weireld and G. Maurin, *Angew. Chemie - Int. Ed.*, 2013, **52**, 10316–10320.

S5 P. Zhao, N. Liu, C. Jin, H. Chen, Z. Zhang, L. Zhao, P. Cheng and Y. Chen, *Inorg. Chem.*, 2019, **58**, 8787–8792.

S6 J. M. Taylor, T. Komatsu, S. Dekura, K. Otsubo, M. Takata and H. Kitagawa, *J. Am. Chem. Soc.*, 2015, **137**, 11498–11506.
